# A SiO_2_ Hybrid Enzyme-Based Biosensor with Enhanced Electrochemical Stability for Accuracy Detection of Glucose

**DOI:** 10.1155/2023/6620613

**Published:** 2023-06-02

**Authors:** Li Mei, Yiting Yang, Jiagen Li, Shuyong Shang, Xiaohong Fu

**Affiliations:** ^1^Department of Culinary, Sichuan Tourism University, Chengdu 610100, China; ^2^Provincial Key Laboratory for Structural Optimization and Application of Functional Molecules, Chengdu Normal University, Chengdu 611130, China

## Abstract

A novel enzyme-based biosensor for glucose detection is successfully developed using layer-by-layer assembly technology. The introduction of commercially available SiO_2_ was found to be a facile way to improve overall electrochemical stability. After 30 CV cycles, the proposed biosensor could retain 95% of its original current. The biosensor presents good detection stability and reproducibility with the detection concentration range of 1.96 × 10^−9^ to 7.24 × 10^−7^ M. This study demonstrated that the hybridization of cheap inorganic nanoparticles was a useful method in preparing high-performance biosensors with a much lower cost.

## 1. Introduction

Diabetes mellitus is the most prevalent chronic disease, which always comes with abnormally high blood glucose levels [1]. On-time monitoring of the glucose level in the blood and body fluids could present useful information in the diabetes mellitus treatment. But the complex chemical and biological environment in the body fluids and blood presents a huge challenge for accurate direct detection of glucose. Since the first report of glucose biosensors by Clark and Lyons in 1962, enzyme-based biosensors have been widely studied and developed [2]. Electrochemical biosensors possess numerous advantages in the rapid and accurate detection. Also, the co-use of enzymatic reactions further improves the selectivity of the biosensor [3].

Glucose oxidase (GOx) is the most commonly used enzyme in glucose detection due to its high selectivity and rapid response. For GOx-based biosensor, the loading amount of GOx plays an important role in the detection accuracy and sensitivity. However, the good water solubility of GOx might lead to the leaching of GOx during electrochemical detection and further lead to poor performance in detection accuracy and reproducibility [4]. Thus, attempts have been made to achieve effective GOx immobilization by the introduction of an electrode matrix. Several materials have been explored for the efficient loading of the GOx, including carbon nanotubes (CNTs), polyaniline, graphene, and metal nanoparticles [5–8]. Among these, CNTs show great potential for enzyme loading due to their large surface area. However, despite the good conductivity of the CNTs themselves, the GOx immobilized electrode matrix still suffers from poor conductivity, which results in poor electrochemical properties [9]. Thus, materials, such as gold nanoparticles (AuNP) and the thionine (THi), are always doped into the electrode matrix for better conductivity [10].

Multicomponents electrode matrix presents great application potential, but the formation of a stable electrode matrix is still a challenge. Chemical crosslinking of different components in the electrode matrix could improve physical stability but raise costs and lower fabrication convenience. Also, the introduction of extra reaction reagents might alter the electrochemical properties [11]. Thus, a better way of constructing high-performance biosensors with high stability is still needed.

Here, a novel electrochemical glucose biosensor (SiO_2_-CNTs/THi/AuNPs/GOx) with good stability was fabricated through noncovalent interaction with layer-by-layer assembly technology. CNTs were used as supporting materials, and SiO_2_ hybrid was applied to improve the stability of the multi component electrode matrix. Thionine and AuNP were also introduced for enhanced conductivity. The role of the SiO_2_ nanoparticles in the formation of the electrode matrix was studied, and enhanced stability was found by electrochemical analysis. The biosensor demonstrated that the SiO_2_ hybrid was a novel and convenient method to produce high-performance glucose biosensors.

## 2. Materials and Methods

### 2.1. Chemicals

SiO_2_ was purchased from Degussa AG, Germany. GO was purchased from Solarbio, Beijing. Glucose was from Jinshan Chemical Test, Chengdu. CNTs dispersion in N-methyl-2-pyrrolidone (NMP) was obtained from Chengdu Organic Chemicals co., Ltd. Chinese academy of sciences, Chengdu. Chloroauric acid (HAuCl4) was purchased from Sinopharm Chemical Reagent co., Ltd, Shanghai. Thionine was from Shanghai Yuanye Bio-technology Co., Ltd, Shanghai. All other reagents and solvents were of analytical grade and commercially available and used without further purification. Ultra-pure water (18.25 MΩ) was used throughout.

### 2.2. Preparation of SiO_2_-CNTs/THi/AuNPs/GO Biosensors

Firstly, pretreat the glassy carbon electrode (GCE). GCE (*φ*3 mm) was first polished using Al_2_O_3_ (*φ*0.3 *μ*m) and then with Al_2_O_3_ (*φ*0.05 *μ*m) to be the mirror surface. Then, the electrode was washed with the ethanol and ultra-pure water for 3 times (10 minutes each time) in the sonicator. Then, a cyclic voltammetry (from −0.6 V to 1.0 V in 0.1 M·H_2_SO_4_ solution) was performed to activate the GCE.

Secondly, prepare the SiO_2_-CNTs dispersion and AuNP dispersion. The SiO_2_-CNTs dispersion in NMP was prepared by mixing SiO_2_ with CNTs. The process can be done as follows: the SiO_2_ powder (0.3 mg) was added to a CNTs solution in NMP (1.5 wt%, 7 *μ*L) and then the ultra-pure water (1 mL) was added. The dispersion was further sonicated for 2 h to ensure the even distribution. The gold nanoparticle (AuNP) dispersion in water was prepared by reducing HAuCl_4_ with sodium citrate, as reported before [12]. A typical procedure can be done as follows: HAuCl_4_ (0.0100 g) was first dissolved in 100 mL ultra-pure water and then heated up to 100°C with vigorous shaking. The solution was then refluxed for 15 min, and a solution of sodium citrate (1.00 wt% in water) was added till the dispersion turned gray (approximately 0.8 mL was consumed). The solution was further refluxed till the color turned claret-red and then the dispersion was cooled to room temperature. The final AuNPs dispersion (approximately 0.01 wt%) was stored at 4°C.

**Figure 1:**
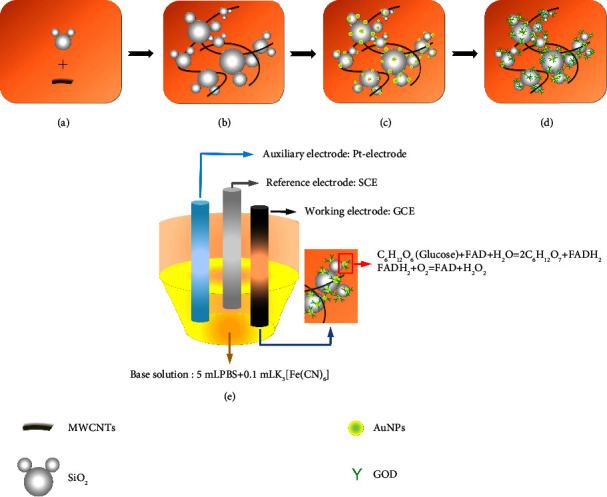
The preparation of SiO_2_-CNTs/THi/AuNPs/GO biosensor.

Thirdly, fabricate the SiO_2_-CNTs/THi/AuNPs/GO biosensor. The proposed electrode was prepared by repeatedly doping multicomponent onto the GCE in order (Figure 1). SiO_2_-CNTs dispersion (4.00 *μ*L), thionine solution (4.00 *μ*L, 0.01 M in water) and AuNP dispersion (4.00 *μ*L) were separately assembled onto the GCE. After each modification, the electrode was stored at 4°C for 4 h to vaporize the solvent. The modification of GO was accomplished with a saturated phosphate buffer solution (PBS) of GO with the same procedure.

### 2.3. Electrochemical Measurements

Electrochemical measurements were performed on a CHI 760E electrochemical workstation (Shanghai Chenhua Instruments Limited, China) with a conventional three-electrode system that consisted of a saturated calomel electrode (SCE) as the reference electrode, a platinum wire as the counter electrode, and a bare or modified GCE as the working electrode.

**Figure 2:**
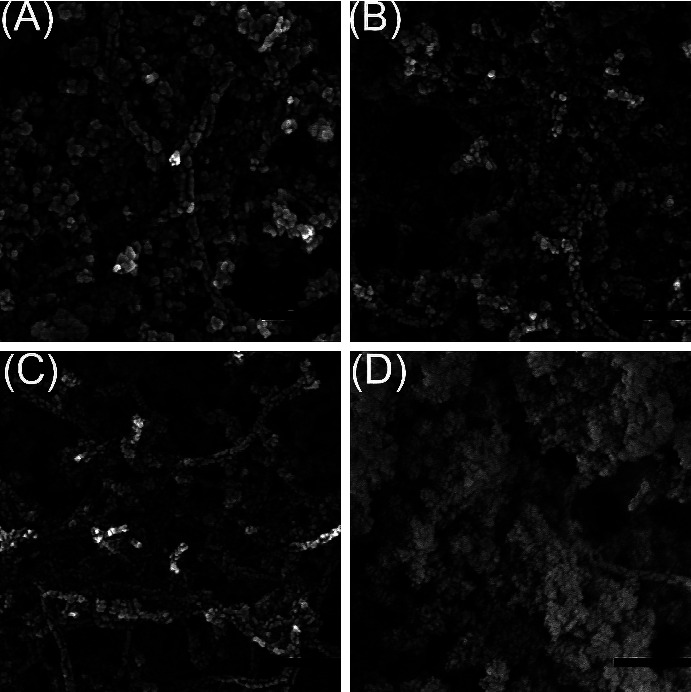
SEM images of (A) SiO_2_-CNTs, (B) SiO_2_-CNTs/THi, (C) SiO_2_-CNTs/THi/AuNPs, and (D) SiO_2_-CNTs/THi/AuNPs/GO composites at the same magnification. The scale bar represents 200 nm.

The detection of glucose via cyclic voltammetry (CV) was performed in PBS (5.00 mL, 0.05 M, pH 7.00) or PBS containing K_3_[Fe(CN)_6_] (5.00 mL, 0.05 M, pH 7.00, concentration of K_3_[Fe(CN)_6_] was 0.02 M) with a scanning rate of 100 mV/s (from −0.6 V to 1.0 V) at 25 ± 0.5°C (Figure 2). AC impedance was employed to characterize the modification process of the biosensor with a frequency range of 1 to 105 Hz and an amplitude of the AC potential of 5 mV.

For the detection of glucose, a concentration-current working curve was first acquired by adding a predetermined amount of glucose solution into the electrochemical cell. The concentration of glucose can be calculated from the current at oxidation peaks using the working curve.

### 2.4. Analysis and Characterization

The morphology of the samples was characterized with a Zeiss sigma 300 scanning electron microscope (SEM). Samples were firstly doped onto GCE as described above. Then the samples for each step were gently removed from GCE, collected, and used for SEM tests directly.

The FT-IR spectroscopy analysis was performed on a Nicolet Satellite infrared spectrometer in the range 400–4000 cm^−1^ with a resolution of 4 cm^−1^ using KBr pellet technique.

## 3. Results and Discussion

### 3.1. Biosensor Fabrication

The glucose-sensitive biosensor was formed by the composition of several components on GCE. The proposed biosensor utilizes GO to achieve specific glucose detection. The immobilization capacity of enzymes plays an important role in detection accuracy and reproducibility. To maintain the flexibility of biosensor production and enhance the reliability of enzyme loading, commercially available SiO_2_ nanoparticles were introduced into the layer-by-layer assemble process. Also, the multicomponent electrode matrix, containing thionine and gold nanoparticles, was applied to the GCE to fulfil the requirements of sensitive and accurate glucose detection.

#### 3.1.1. Layer-By-Layer Preparation of the Biosensor

**Figure 3:**
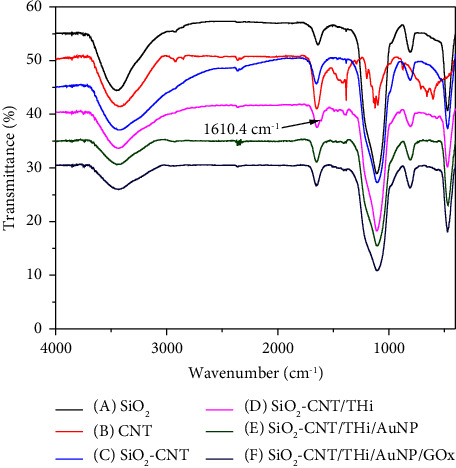
FTIR spectra of (A) SiO_2_, (B) CNT, (C) SiO_2_-CNTs, (D) SiO_2_-CNTs/THi, (E) SiO_2_-CNTs/THi/AuNPs, and (F) SiO_2_-CNTs/THi/AuNPs/GOx composites.

**Figure 4:**
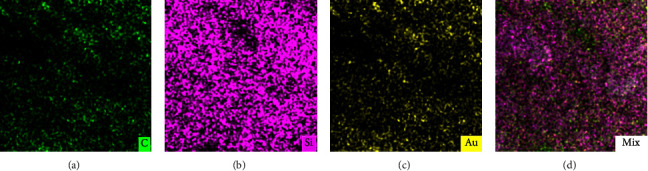
EDS images of SiO_2_-CNTs/THi/AuNPs/GOx. Distribution of C (a), Si (b), and Au (c) were presented separately and mixed images (d). The scale bar represents 200 nm.

The biosensor was prepared by doping the different composites onto GCE, and the successful modification of each step was confirmed by SEM (Figure 2) and FT-IR (Figure 3) technology. SiO_2_-CNTs complex was first introduced onto GCE. As shown in Figure 2(a), no obvious self-aggregate of SiO_2_ nanoparticles can be seen. On the contrary, SiO_2_ nanoparticles were assembled onto CNTs. This phenomenon provides the possibility of SiO_2_ acting as a crosslinker. The follow-up modification of thionine and gold nanoparticles leads to no obvious morphology changes (Figures 2(a)–2(c)), thus FT-IR and EDS were used to confirm the existence of thionine and gold nanoparticles, as shown in Figures 3 and 4. The appearance of a peak at 1610.4 cm^−1^ confirms the successful introduction of thionine molecular, while the yellow color of Au in EDS tests, as shown in Figure 4 confirms the existence of AuNPs in the final biosensor. After the addition of GO, large aggregates can be witnessed (Figure 2(d)). GO was introduced to achieve selective glucose detection.

**Figure 5:**
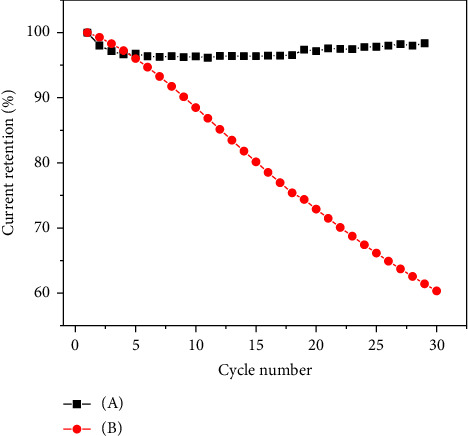
Stability of different biosensors. (A) SiO_2_-CNTs/THi/AuNP/GOx biosensor; (B) CNTs/THi/AuNP/GOx biosensor. The current was recorded by CV test in PBS K_3_[Fe(CN)_6_] at 0.075 V.

The addition of SiO_2_ was essential in this experiment. Pre-experiments (data not shown) found that without the addition of SiO_2_, the modification of the GCE with the SiO_2_-CNTs/THi/AuNPs/GO composites was not physically stable enough, and the composite might fall off from the GCE during the electrochemical experiments. Also, the unstable formation of the electrode matrix might result in the loss of enzyme during the electrochemical test, which further leads to a poor electrochemical stability, as shown in Figure 5. The strong interaction between CNTs and SiO_2_ nanoparticles made the modification of GCE through the dripping method much more effective and greatly helped to glue the composite onto GCE. By mixing SiO_2_ nanoparticles with the NMP solution of CNTs, the SiO_2_ was first absorbed onto the CNTs without obvious self-aggregation of SiO_2_ (Figure 2(a)). Further introduction of different composites (Figures2(b), 4 (c), 4 (d)) happened on the SiO_2_ nanoparticles, thus providing the “crosslink” effect of SiO_2_.

### 3.2. Electrochemical Characterization

**Figure 6:**
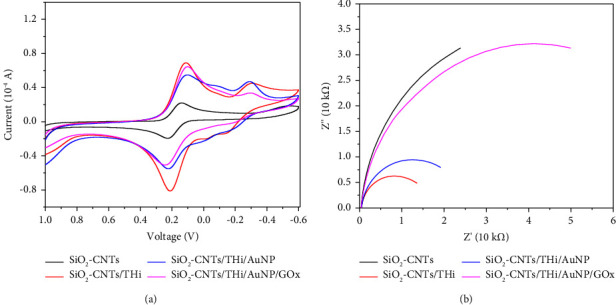
(a) The CV of electrode with different modifications (in PBS with 0.02 M K_3_[Fe(CN)_6_]) and (b) the AC impedance of electrode with different modifications.

CV and AC impedance were also performed in PBS (0.05 M, pH = 7.00 with 0.02 M K_3_[Fe(CN)_6_]) to study the effects of modification on the electrochemical properties of the electrode. As shown in Figure 6, only the redox peak of K_3_[Fe(CN)_6_]) can be seen for SiO_2_-CNTs (Figure 6(a)) electrode with a high impedance value (Figure 6(b)). After introducing thionine, the strong redox peaks of thionine can be found at −0.4 V and 0.1 V (Figure 6(b)), which confirms the existence of thionine. The introduction of thionine also decreased the impedance value (Figure 6(b)), due to the excellent electron transfer efficiency of thionine [13]. After the modification of gold nanoparticles (Figure 6(a)), the peak current decreased while the impedance value increased slightly. We suggested this phenomenon might be caused by the thiol-gold interaction formed between AuNP and -SH in thionine, which limits the electron transfer ability of thionine. Further modification of GOx leads to an obvious increase in impedance value (Figure 6(b)) since the GOx exhibits poor electroconductivity.

### 3.3. Optimization of Test Condition

The accurate detection of glucose heavily relies on the activity of the GOx modified on the electrode. Thus, to maximize the activity of the GOx and the sensitivity and accuracy of the detection, temperature and pH during the CV test were, respectively, optimized.

**Figure 7:**
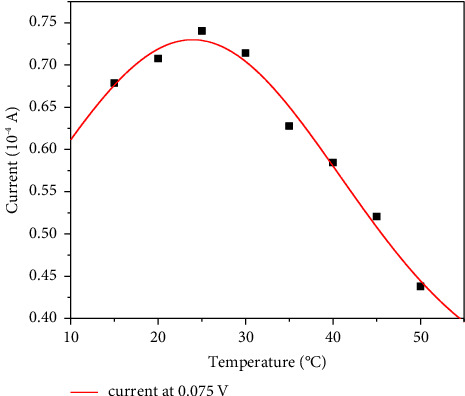
Peak current at 0.075 V at different temperatures.

As shown in Figure 7, the current at 0.075 V reaches the maximum value (0.75 × 10^−4^ A) when the CV test is performed at 25°C, which indicates this temperature favors the activity of the GOx. This phenomenon was consistent with the literature report before. Lower temperatures inhibited the activity of GOx and led to a smaller current, while high temperatures caused irreversible damage to the enzyme. Thus, 25°C was selected as a favorable temperature for this biosensor in future experiments.

**Figure 8:**
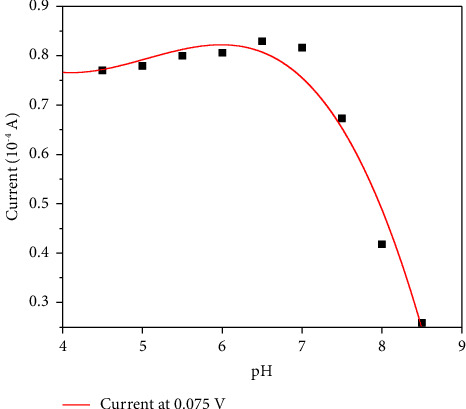
Peak current at 0.075 V at different pH.

During the optimization of pH, different phenomena were found. According to Figure 8, the biosensor shows a similar sensitivity to glucose in a wide pH range from 4.50 to 7.00, indicating this biosensor might be suitable for a variety of samples from weak acids to neutrals. The peak current decreased dramatically when pH higher than 7.00, suggesting that the enzyme might be unstable in base. Thus, 7.00 was selected as favorable pH for this biosensor in future experiments.

### 3.4. Stability of the Biosensor

To confirm the effect of SiO_2_ as a crosslinker, CV test of 30 CV cycles was conducted to study its influence on stability (Figure 5). For the biosensor without the SiO_2_ hybrid, the current retention rate was about 60% after 30 CV cycles. While the addition of SiO_2_ resulted in a 96% current retention after 30 cycles with a current loss of less than 5%. The much-improved long-term stability might result from the enhanced physical stability of the electrode matrix, as suggested from the SEM images above, in which SiO_2_ acts as a crosslinker.

### 3.5. Working Curve and Determination of Glucose

**Figure 9:**
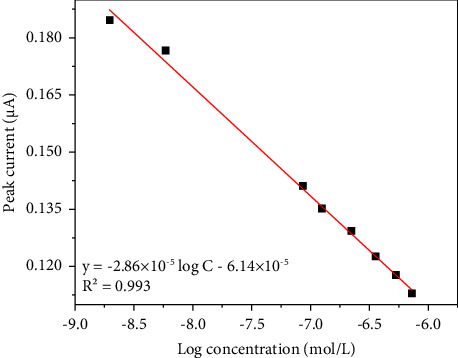
Working curve for the detection of glucose.

Figure 9 shows the relation between the concentration of glucose and the peak current in which the concentration of glucose is between 1.96 × 10^−9^ and 7.24 × 10^−7^ M. The regression equation is *y* = −2.86 × 10^−5^ log C–6.14 × 10^−5^ (*R*^2^ = 0.993), which presents a good linear relationship between concentration and peak current.

The detection accuracy and precision of the proposed electrode were further analyzed through the detection of preweighed samples under the optimized condition. The accuracy was described as the mean relative error. [14] For a sample with a glucose concentration of 1.92 × 10^−8^ M, the biosensor suggests a concentration of 1.82 × 10^−8^ M, with an average detection recovery rate of 105% and accuracy of 5% (*n* = 5). This affirms that the proposed electrode could present an accurate detection toward glucose.

## 4. Conclusions

In summary, a novel glucose biosensor was successfully fabricated using layer-by-layer assembly technology. SiO_2_ was introduced into the electrode matrix as the physical crosslinker. Electrochemical analyses were carried out to confirm the enhanced stability. The improved physical stability of the electrode matrix promoted GOx immobilization and further ensured accurate, sensitive, and selective glucose detection with a detection range from 1.96 × 10^−9^ to 7.24 × 10^−7^ M. This work demonstrated a novel way to fabricate an effective and efficient electrochemical biosensor through the hybridization of cheap inorganic components, which provides a new possible solution to bridging the gap between laboratory and practical applications of electrochemical analysis.

## Data Availability

The TXT data used to support the findings of this study are included within the supplementary information files.

## Supplementary Materials

Supplementary Materials The first supplementary material file is the original data of the CV and AC impedance of electrode with different modification. The second supplementary material file is the original data of the peak current at different temperatures and pH. The third supplementary material file is the original data of the working curve for the detection of glucose.
